# Randomized Pilot Study of 20 Gy in 5 Fractions versus 27 Gy in 3 Fractions Radiotherapy for Treating Painful Bone Metastases: A Single Institution Experience

**DOI:** 10.31557/APJCP.2020.21.6.1807

**Published:** 2020-06

**Authors:** Amr Sakr, Wedad Bassam Hashem, Nadia Ebrahim, Karim Nabil Mashhour

**Affiliations:** *Department of Clinical Oncology, Kasr Al-Einy School of Medicine, Cairo University, Egypt. *

**Keywords:** Palliative radiotherapy, bone metastases, Stereotactic body radiosurgery, hypofractionation

## Abstract

**Purpose:**

Radiotherapy is a very effective tool in the treatment of painful bone metastases. The aim of this study was to compare the palliative effect of radiotherapy between the standard fractionation schedule 20 Gy over 5 fractions (20Gy/5fr) and the high biological dose schedule 27 Gy over 3 fractions (27Gy/3fr) which is frequently used in Stereotactic body radio-surgery (SBRT).

**Methods:**

Patients were randomized to receive (20Gy/5fr)or (27Gy/3fr). The primary aim of the study was pain relief using the numeric rating scale (NRS), after three months of radiation therapy. Secondary end points include pain relief immediately after finishing radiation therapy (within one week), and narcotic relief after three months of radiation therapy.

**Results:**

Twenty-two patients with painful bone metastases were included. 12 patients received (20Gy/5fr) and 10 patients received (27Gy/3fr). Male patients were predominant on both arms (81.8%) with a mean age of 58 years [ranging between 19-72 years]. For pain relief after three months of radiation therapy, partial pain relief was documented in 9 patients (75%) with (20Gy/5fr) and in 8 patients (80%) with (27Gy/3fr) with a p- value of 0.6. Additionally, narcotic relief after three months was equal for both groups. For immediate pain relief, partial pain relief was seen in one patient (8%) with (20Gy/5fr) versus seven patients (70%) with (27Gy/3fr) with a p value of 0.06. The increase in immediate pain relief in the 27Gy arm was numerically but not statistically significant.

**Conclusion:**

SBRT and standard fractionation radiation therapy had equal effectiveness for pain relief, when the assessment was done after three months of radiation therapy. Interestingly, SBRT had a better immediate pain relief.

## Introduction

Painful bone metastasis is a major problem for cancer patients. In addition to pain, the high incidence of presence of skeletal related events like pathological fracture, spinal cord compression and hypercalcaemia can lead to major disability of cancer patients. Data from lung and breast cancer patients proved that patients with bone metastases had a shorter overall survival than patients without bone metastases. (Kuchuka et al., 2015; Schröder et al., 2017).

External beam radiation therapy is one of the main lines of palliative treatment of bone metastases; it can provide 50-70% partial pain relief and 15-25% complete pain relief. Different dose schedules are used, ranging from 40 Gy over 20 fractions, 30 Gy over 10 fractions, 20 Gy over 5 fractions and only a single fraction 8 Gy. Published data proved that all had an equivalent palliative effect. However, the use of a single-fraction was associated with more pathological fractures and the need for re-irradiation. (Steenland et al., 1999; Hartsell et al., 2005; Bone Pain Trial Working Party 1999; Chow et al., 2007)

On the other hand, new data support the use of stereotactic body radiotherapy (SBRT) for the palliation of bone metastases. With this technique, we can deliver high radiation dose per fraction. It is safe, achieves good tumor control and palliation of pain. The dose of radiation therapy in SBRT was also variable, ranging from 24 Gy in a single fraction, 27 Gy in three fractions, or 30 Gy in five fractions. (Nguyen et al., 2010; Wang et al., 2012; Habl et al., 2017)

Accordingly, we have two different approaches; the traditional radiation therapy or the high biological dose using SBRT. We planned to do a pilot randomized phase II trial comparing both approaches. We chose 20 Gy over 5 fractions as the best suitable schedule for the traditional approach and 27 Gy over three fractions for SBRT approach. In order to know which is more beneficial for our patients, we chose pain relief at three months as the primary end point.

## Materials and Methods

The study protocol was approved from the center scientific committee. It was a prospective randomized phase II pilot study comparing 20 Gy over 5 fractions versus 27 Gy over 3 fractions, as a different schedule of radiation therapy for painful bone metastasis.

*Patient population*

Twenty-two patients were recruited according to inclusion and exclusion criteria. Patients were eligible if they were 18 years of age or older and had histologically proven primary malignancy and radiographic evidence of bone metastasis. For example, in the case of spinal metastasis, the presence of spinal instability or neurological deficit may preclude patients from the study as the results may be biased by the surgical treatment required. A maximum of three distinct bone metastases were allowed. Karnofsky performance status of at least 40 was allowed. A Lesion less than 5mm from spinal cord may make SABR regimens difficult to apply due to danger of exceeding spinal cord tolerance. The main exclusion criteria were previous radiation therapy to the affected painful site, pathologic fracture or impending fracture of the treatment site, if there was a clinical or radiographic evidence of spinal cord or cauda equine compression. Haematological tumours are excluded from the study since they are more radiosensitive and respond well to lower doses per fraction and total doses. Patients receiving bone-supporting drugs such as Bisphosphonates or denosumab were included.

*Aim of the study*

The aim of this study was to compare the pain-relieving efficacy of 20 Gy in 5 fractions versus 27 Gy in 3 fractions in patients with painful bone metastases. The primary aim of the study was pain relief using numeric rating scale (NRS) after three months of radiation therapy. Secondary end points include pain relief one week after finishing radiation therapy, narcotic relief after three months of radiation therapy.

*Pain assessment*

Baseline pain assessment by using a numerical rating scale from 0 to 10 where 0 indicates no pain and 10 indicates severe pain. Patients rated their pain from 0 to 10 prior to start of radiation therapy. The score is recorded along with the analgesic used and its dose. 

*Radiation therapy steps and technique*

*Radiation therapy technique was done by the following steps*

*CT scan*

CT scanning was carried out with the patient lying comfortable and supine. Midline and lateral laser lines were used for target localization, with radio-opaque markers being utilized for visualization of the reference points on the CT image. Centres were tattooed for reproducibility. Images were then transferred to the treatment planning system (Eclipse version 11). 

*Contouring*

Gross tumor volume (GTV) was delineated guided by available diagnostic imaging modality (Bone scan, MRI or PET scan) and a margin of 0.3cm (Planned tumor volume or PTV) was taken around the GTV. Risk structures were contoured according to the site of the bone lesion indicated for radiation therapy.

*Planning*

**Figure 1:**
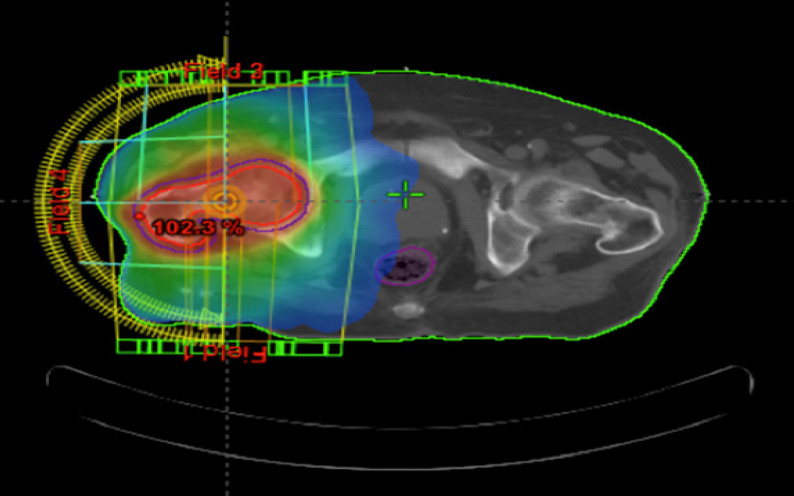
VMAT Planning Technique for a Case of Metastatic Cancer Breast to the Neck of Femur

Patients were planned either by 3D conformal or VMAT modalities, figure (1) demonstrate a case planned with VMAT. Dose- volume histogram was then used to calculate the normal tissue dose distribution (DVH).

*CT simulation*

Virtual CT simulation is done before start of treatment guided by the tattoo done earlier and the Digital reconstructed radiograph (DRR).

*Verification*

**Figure 2:**
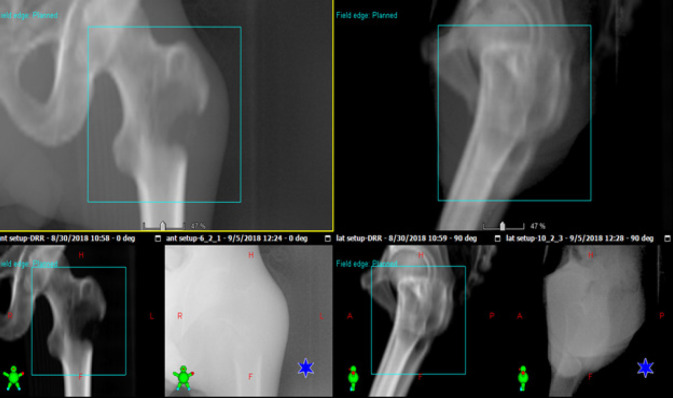
Online EPID for a Ccase of Metastatic Cancer Prostate to Bone

On the treatment machine daily online Electronic portal image device (EPID) was done to ensure proper positioning of the patients, as seen in figure (2).

*Treatment given and Follow up*

Patients in the (20Gy/5fr) schedule received their fractions on five consecutive days. While in (27Gy/3fr) schedule, they received fractions on an every other day basis. After finishing the radiation treatment and after three months period, patients were asked to score their pain using NRS and the type of analgesic used and its dose was noted.

*Response categories*

*Pain relief can be classified into one of four categories.*

1) Complete pain relief: a pain score of 0 by NRS at the treated site with no increase in analgesic intake in comparison to baseline analgesic intake.

2) Partial pain relief: a pain score reduction of 2 or more from baseline by NRS at the treated site without increasing analgesic intake, or analgesic reduction of 25% or more from baseline without an increase in pain.

3) Stationary pain relief: any response that is not categorized as complete, partial pain relief, or pain progression.

4) Pain progression: increase in pain score of 2 or more above baseline by NRS at the treated site, or an increase of 25% or more in analgesic intake compared with baseline.

*Statistical Methods*

Data were collected, tabulated and statistically analyzed using a personal computer with (SPSS) version 22 program. (IBM SPSS Statistics for Windows, Version 22.0. Armonk, NY, USA: IBM Corp). P value of ≤ 0.05 was considered statistically significant. Chi-square test was used to assess whether the distribution of a categorical variable is significantly different between two or more groups. Tests of chi-square were used to determine whether there is an association between two categorical variables and used to detect any difference between the two test groups. The Wilcoxon test was used to test the null hypothesis that the median of the differences between pairs of observations is equal to zero.

## Results

This pilot study was carried out in Kasr Al-Ainy Center of Clinical Oncology and Nuclear medicine (NEMROCK) during the period between May 2018 and January 2019.Twenty-two patients with painful bone metastases were included. Twelve patients received (20Gy/5fr) and 10 patients received (27Gy/3fr). Male patients were predominant on both arms (81.8%) with a mean age of 58 years [ranging between 19-72 years]. Only two patients in (20Gy/5fr) and one patient in (27Gy/3fr) were on chemotherapy. Different baseline characteristics of both groups are illustrated in Table (1). 

*Pain relief*

All 22 patients underwent pain relief assessment immediately and after three months of radiation therapy. Complete pain relief was not documented in any patient in both groups. Partial pain relief after three months was comparable with a p-value of 0.6. Immediate partial pain relief was seen in seven patients (70%) of 27Gy/3fr schedule versus only one patient (8%) in 20Gy/5fr schedule with a p-value of 0.06. The increase in immediate pain relief in the 27Gy arm was numerically but not statistically significant. Pain relief after 3 months of radiation therapy and immediate pain relief are illustrated in Tables 2 and 3, respectively.

Although pain severity is significantly decreased in both arms, the need for analgesia remained the same for both groups when assessment was done three months after radiotherapy with the exception of one patient who developed pain flare and increased the dose of analgesics. Regarding the possible confounders as age, sex, being on active treatment and receiving bone supporting agents, all these factors did not affect the significance of pain score reduction with p- values 0.7, 0.1, 0.67 and 0.66 respectively which excluded these factors as confounders in both groups.

**Table 1:** Illustrates the Patient and Tumoral Characteristics of Both Arms Involved in the Study

Characteristic	(20Gy/5fr)	(27Gy/3fr)
Number of patients	12	10
Age (years)		
Median	58	58
Range	19-70	35-72
Sex		
Male	12	6
Female	0	4
Primary		
Prostate	1	3
Bladder	1	1
Breast	0	1
Hepatocellular	3	1
Thyroid	1	0
Sarcoma	1	2
Colon	1	0
Nasopharynx	1	0
Maxilla	0	1
MUO	3	1
Active treatment		
Chemotherapy	2	1
Hormonal	0	1
Bone supporting agents	2	4
Baseline Pain		
Range	4-10	4-10
Median	6	8

**Table 2:** Pain Relief after 3 Months of Radiation Therapy

	Dose and Fractions		
Variable	(20Gy/5fr) No. (%)	(27Gy/3fr) No. (%)	*P*-value
Complete pain relief	0 (0)	0(0)	-
Partial pain relief	9 (75)	8(80)	0.6
Stationary pain relief	3 (25)	1(10)	-
Pain progression	0(0)	1(10)	-

**Table 3:** Illustrates Immediate Pain Relief after Receiving Treatment

Variable	(20Gy/5fr) No. (%)	(27Gy/3fr) No. (%)	*P*-value
Complete pain relief	0 (0)	0 (0)	-
Partial pain relief	1 (8)	7 (70)	0.06
Stationary pain relief	11 (92)	3 (30)	-
Pain progression	0 (0)	1 (10)	-

## Discussion

This is a pilot prospective randomized phase II study for patients with painful bone metastasis, between two different radiotherapy schedules, 20Gy/5Fr versus 27Gy/3Fr. Accordingly, it is a comparison between the traditional palliative dose of radiation therapy using 2D or 3D techniques 20Gy/5Fr and the newly developed high biological dose, which is recently used by SBRT technique 27Gy/3Fr. Our question in this study was very clear and simple, which schedule is better for our patients. We used pain control after three months of radiation therapy as the primary end point. There is no other parameter to tell us which is better; except the patients themselves. So, we depend on NRS in pain relief assessment. 

We have selected pain relief after three months of radiation therapy as the primary end point, like many previous trials including The Radiation Therapy Oncology Group (RTOG) trial 97-14 (Steenland et al., 1999; Hartsell et al., 2005), as it is the most reliable, simple and clear objective. Interestingly, we found that immediate pain relief after just finishing radiation therapy is very important as the primary aim was palliation, and the timing of palliation is very crucial for our patients. 

Our study documented that immediate pain relief was evidently better in (27Gy/3fr) schedule. However, both groups are equally effective according to the pain assessment that was done three months after the radiation therapy. Thus, we reached a conclusion that high biological dose can provide faster pain relief. This prompts the recommendation of performing future trials with larger study populations to confirm this finding. 

Other important parameters that should be taken into consideration include; cost of the treatment, time taking for delivery of radiation therapy, availability of the new machines and the palliative aim of treatment. All these parameters are in favor with the use of traditional schedules of radiation therapy. Accordingly, in order to apply SBRT we have to have significant better results in comparison to the traditional techniques. 

Delivering SBRT with high dose per fraction using sophisticated techniques in radiation therapy requires meticulous care starting from the patient selection, fixation technique up to the daily verification methods. This raised a point that using SBRT for pain control is equally as effective as traditional radiotherapy techniques and hence to control pain symptom is not essential.

We have not reported any case with complete pain relief, which opposes what was documented in the literature of approximately 15-25% complete pain relief (Steenland et al., 1999; Hartsell et al., 2005; Bone Pain Trial Working Party 1999; Chow et al., 2007). The explanation is that most of our patients are metastases from relatively radio-resistant primary tumors, hepatocellular carcinoma, sarcoma, and hormone-refractory prostate cancer. Additionally, most of our patients are not receiving concomitant active treatment. We believe that the small sample size may be a contributing factor.

Almost all the patients remained on the same dose of analgesics after the 3 months possibly because no patient attained complete response based on our assessment and criteria except for one patient who developed pain flare and increased the dose of analgesics. Again, it can be explained by the type of patients and the relatively no concomitant active treatment.

There was no statistically significant correlation found between age, sex, receiving bone-supporting agents, being on active treatment and response to pain possibly due to the low sample size. However, you can see the real palliative effect of radiotherapy when you do not give concomitant active treatment.

The main limitation of this study is the limited number of patients. However, up to our knowledge this is the first study to do this comparison. In the near future, we are going to do this trial over a large number of patients in order to know which group of patients, which can benefit from high biological dose. And we can select one or two primary tumor e.g. breast or lung cancer, in order to get reliable results. A possible recommendation for larger studies in the future with longer follow-up may be the use of skeletal-related events occurrence (e.g. pathological fractures, spinal cord compression, hypercalcemia) as an assessment tool comparing the utility of both fractionation schedules.

In conclusion, although, we had an immediate better pain relief with SBRT. We did not find any difference in pain relief after three months of radiation therapy between SBRT and traditional palliative schedules. The traditional palliative schedules should continue to be the practice of our daily life in the treatment of painful bone metastases.
